# Tumor-derived cell-free DNA and circulating tumor cells: partners or rivals in metastasis formation?

**DOI:** 10.1007/s10238-023-01278-9

**Published:** 2024-01-17

**Authors:** Andréa Witz, Julie Dardare, Margaux Betz, Pauline Gilson, Jean-Louis Merlin, Alexandre Harlé

**Affiliations:** Département de Biopathologie, Institut de Cancérologie de Lorraine, CNRS UMR 7039 CRAN-Université de Lorraine, 6 avenue de Bourgogne, 54519 Vandœuvre-lès-Nancy Cedex, France

**Keywords:** Metastasis, cfDNA, CTCs, Metastatic cascade, Genometastasis

## Abstract

The origin of metastases is a topic that has sparked controversy. Despite recent advancements, metastatic disease continues to pose challenges. The first admitted model of how metastases develop revolves around cells breaking away from the primary tumor, known as circulating tumor cells (CTCs). These cells survive while circulating through the bloodstream and subsequently establish themselves in secondary organs, a process often referred to as the “metastatic cascade”. This intricate and dynamic process involves various steps, but all the mechanisms behind metastatic dissemination are not yet comprehensively elucidated. The “seed and soil” theory has shed light on the phenomenon of metastatic organotropism and the existence of pre-metastatic niches. It is now established that these niches can be primed by factors secreted by the primary tumor before the arrival of CTCs. In particular, exosomes have been identified as important contributors to this priming. Another concept then emerged, *i.e.* the “genometastasis” theory, which challenged all other postulates. It emphasizes the intriguing but promising role of cell-free DNA (cfDNA) in metastasis formation through oncogenic formation of recipient cells. However, it cannot be ruled out that all these theories are intertwined. This review outlines the primary theories regarding the metastases formation that involve CTCs, and depicts cfDNA, a potential second player in the metastasis formation. We discuss the potential interrelationships between CTCs and cfDNA, and propose both in vitro and in vivo experimental strategies to explore all plausible theories.

## Introduction

Metastasis stands as the primary contributor to cancer-related deaths, embodying an evolutionary process that starts with the primary tumor and progresses to an aggressive, systemic disease. Metastatic subclones can manifest early or late in the primary tumor's existence, yet the precise mechanisms governing their emergence remain enigmatic [1].

The accepted stages of metastasis encompass cell detachment from the primary tumor, intravasation into the circulatory or lymphatic systems as circulating tumor cells (CTCs), survival in the circulation (via hematogenous and/or lymphatic routes), arrest and homing at a distant organ site, extravasation, viability within a new environment, and ultimately, metastatic colonization [2]. A second model, the “mechanical-circulatory” model proposed by Ewing, challenges the “seed and soil” theory, highlighting mechanical factors such as vascular and lymphatic flow to explain the dynamics of tumor metastasis [3].

As far back as the 1930s, Griffith et al. employed a Pneumococcal model to describe for the first time the term “transformation”, delineating the process which we now recognize [4]. In 1944, Avery, MacLeod, and McCarty identified DNA as the “transforming principle” previously described by Griffith [5], pinpointing transformation as a horizontal gene transfer (HGT) wherein DNA encoding malignancy traits transfers from one bacterium to another and incorporates into the recipient genome via homologous recombination (HR) [6]. The term “transfection” is preferred when purified or naked DNA is introduced into an animal cell. Common methods for DNA transfection encompass virus-mediated (biological), chemical, and physical methods [7]. In 1999, Holmgren et al. demonstrated HGT of DNA between cells via the phagocytosis of apoptotic bodies, introducing the conceivable role of cell-free DNA (cfDNA) in metastasis [8, 9].

The intriguing yet controversial hypothesis of “genometastasis” emerged in the 2000s via García-Olmo et al*.* [10]. This notion suggests that metastases might arise from the transfection of susceptible cells in distant target organs with dominant oncogenes from the primary tumor, which circulate in plasma as circulating tumor DNA (ctDNA) [11].

This review aims to describe the main theories surrounding the genesis of metastases involving CTCs and to introduce the cfDNA as a potential second player in the metastasis formation, while unraveling their disparities, potential connections, and proposing strategies to ascertain which of the two holds precedence or if they collaboratively contribute.

## Circulating tumor cells (CTCs)

### Metastatic diffusion

The term "metastasis" first appeared in the nineteenth century, aiming to differentiate between primary and secondary tumors. This term denotes the creation of secondary tumors distant from the initial lesion. In 1970, Fidler et al. outlined the “metastatic cascade” as a model for cancer dissemination, asserting that metastasis unfolds through multiple sequential steps stemming from the primary tumor. All these steps are pivotal in generating a metastasis from a primary tumor [12].

The first step is invasion. Primary tumor cells undergo transformation and gradual growth. By instigating angiogenesis and fostering new capillary networks or leveraging existing ones, neoplastic cells escape from the initial tumor site to establish themselves in other locations [13]. For an extended period of time, the linear progression model was the gold standard of tumor progression, proposing that metastases originated from completely malignant cells, their appearance coinciding with tumor size. Primary tumor growth is progressive, and the dissemination of tumor cells typically aligns with advanced cancers. Interestingly, metastases can also arise from existing metastases. Notably, metastatic spread might occur early in the lifecycle of the primary tumor, referred to as the parallel progression model [14]. Both linear and parallel progression models can coexist within the same patient [1].

Invasion involves a process termed epithelial to mesenchymal transition (EMT), partially driven by transforming growth factor-beta (TGF-β) secreted by cancer-associated fibroblasts (CAFs) within the tumor microenvironment. This process prompts the loss of cell–cell adhesion, facilitates migration and invasion, and confers resistance to apoptosis [15]. Exosomes containing EMT-inducers (TGF-β, hypoxia-inducible factor 1 alpha (HIF1α), and β-catenin) are taken up by recipient cells in the tumor stroma, causing cellular changes, and resulting in facilitated EMT in epithelial cells [16]. Additionally, tumor-associated macrophages (TAM) play a role in EMT induction and subsequent tumor cell invasion [17]. However, while EMT is critical for metastasis invasion, no evidence substantiates EMT in the primary tumor as a precursor to metastasis [18].

Following EMT, tumor cells can infiltrate the stroma and enter the circulation as CTCs. This stage, known as intravasation, takes place in blood vessels and the lymphatic system. Intravasation involves intrinsic factors such as genetic background, epigenetic alterations, metabolism, and mechanical properties, as well as extrinsic factors related to the host tissue and organ microenvironment [19]. Successful angiogenesis and lymphangiogenesis are prerequisites for this metastatic step [15]. Cells can intravasate either as single CTCs or as micro-emboli/clusters of CTCs, contingent upon the type, stage, and location of the primary tumor. CTC-clusters, composed of at least two tumor cells, originating from the primary or metastatic tumor or possibly aggregating intravascularly from single CTCs, exhibit greater metastatic potential and apoptosis resistance compared to single CTCs. Indeed, Fidler et al. indicated that emboli containing five or more tumor cells are more prone to metastasize than single CTCs [20]. CTC-cluster formation involves partial EMT and hypoxia, potentially linked to hypomethylation at critical sites [21, 22]. Although the formation and intravasation mechanisms of CTCs remain incompletely understood, this step is widely admitted.

Within circulation, 63.8% of CTCs perish after 13 days, while 36.1% remain solitary cells, 0.07% endure to establish micrometastases and 0.02% form macrometastases [23]. To achieve this, cancer cells must escape the immune system and avoid elimination by natural killer (NK) cells, which are anti-tumor immune cells. Platelet aggregate formation around CTCs offers protection against NK cells and the shear forces of circulation, as CTCs need to withstand the blood vessel flow rates and shear stresses [15]. The tumor-educated platelets (TEPs) concept suggests that CTCs can educate platelets via secreted mRNA [24]. Platelet RNA analysis has demonstrated 71% accuracy in indicating the location of the primary tumor [25]. Moreover, the survival of CTCs in circulation may be influenced by physiological filters, like the liver as CTCs transit through the portal vein. Single CTCs can evade these filters, unlike CTC-clusters, potentially explaining why single CTCs outnumber CTCs-clusters in fluids [26].

After survival in circulation, cancer cells can arrest in target organs, exit blood vessels, infiltrate host organs, and undergo a shift from mesenchymal to epithelial cells called MET. This transition gives rise to disseminated tumor cells (DTCs) [15]. The extravasation process, or exit from blood vessels, might be facilitated by extracellular vesicles (EVs), particularly exosomes, released by cancer cells. Exosomes can alter endothelial cell cytoskeleton, influencing endothelial barrier permeability [27]. Furthermore, exosomes participate in the formation of TEPs and neutrophil extracellular traps (NETs), both promoting CTCs extravasation [24].

The next step in the metastatic cascade involves DTCs homing to host organs. For this to succeed, the host environment must be suitable to DTCs colonization, proliferation, and macrometastases formation. A portion of DTCs, however, enters dormancy in host organs. This state enhances cell survival and adaptation, with tumor latency lasting from a few weeks to several years, as in lung and prostate cancers, respectively, for example. The transition to dormancy can occur during tumors formation or after DTCs dissemination, enhancing DTCs adaptation to the host organ. Dormant tumor cells may exist as micrometastases or isolated cells [28]. Micrometastases, as dormant tumor masses, sustain latency due to the lack of immune surveillance, reduced blood supply following decreased vascular endothelial growth factor (VEGF) secretion, and apoptosis predominance over proliferation even if cells are still dividing. On the other hand, dormant isolated tumor cells exhibit G0/G1 cell cycle arrest induced by microenvironmental stress factors, this quiescence being reversible. Latency is influenced by the oncogenetic background of the cancer cell, the microenvironment, and the treatment-induced stress, particularly from anti-angiogenic therapies [29]. Transition to the proliferative state depends on the host organ and can be mediated by proliferative signals such as TGF-β1 or periostin [30]. This latency phase constitutes minimal residual disease (MRD), potentially leading to tumor relapse years after curative treatment. MRD denotes the persistence of undetected DTCs following treatment, indicating a poor prognosis due to the high risk of treatment-resistant DTCs recurrence [31]. Notably, the formation of metastasis, whether it is post-treatment or surgery-induced, can be linked to the presence of MRD. Surgery-associated inflammatory responses might enhance CTCs dissemination, fueled by cell proliferation [32, 33]. In certain cancers, such as non-small cell lung cancer (NSCLC), CTCs early dissemination has been identified as the origin of metastatic seeding. CTCs collected post-primary tumor resection shared higher mutation levels with metastases than with the primary tumor, even preceding metastasis onset by months [34]. The latest step of the metastatic cascade entails micrometastasis evolving into macrometastasis.

Furthermore, in addition to forming macrometastases, CTCs can return to the primary site after dissemination and re-seed the original tumor. This phenomenon, coined "tumor self-seeding," demonstrated by Kim et al. [35], results in new mutations among CTCs, creating a new subclones population within the primary tumor [36]. Intriguingly, metastases might release CTC-clusters analogous to the primary tumor, generating monoclonal or oligoclonal metastases [22]. While it remains uncertain whether metastases predominantly comprise CTCs or a subpopulation of more malignant CTCs, primary tumor cells exhibit varied metastatic potentials, forming a heterogeneous cell group. This tumor heterogeneity is potentially explained by clonal evolution (monoclonal, polyclonal, and self-seeding) following the Darwinian model. Microenvironmental changes likely lead to mutation accumulation and epigenetic alterations, fostering tumor subclone emergence and dominant subclone expansion [37]. However, an alternative mutator phenotype model challenges this idea. This model suggests that polyclonal evolution arises from a tumor constituted of numerous small clones, all capable of proliferation [36, 38]. Additionally, Ramaswamy et al. suggested that the complete primary tumor could serve as the metastasis source, rather than a small subpopulation within it [39]. In addition, two other models based on the evolution of cancer stem cells (CSCs) are proposed to explain tumor heterogeneity. The classical CSC model purports that CSCs with the highest tumorigenic potential drive the metastases genesis in a unidirectional fashion, while the plastic CSC model, suggests bidirectional conversion of CSCs to non-CSCs, highlighting the plasticity of CSCs. It should be noted that tumor cells may originate from CSCs or somatic cells [37]. The comparison of driver gene mutations in primary and secondary tumors highlighted shared genetic backgrounds and minimal divergence between them. Notably, functional mutations in driver genes are more clonal than subclonal. These findings support the clonal evolution model as the most suitable model to explain solid cancer progression [36, 40].

### Metastasis organotropism and premetastatic niche

In 1889, Stephen Paget introduced the “seed and soil” hypothesis, a concept addressing the spread of metastases [41]. This model offers an explanatory framework for the emergence and distribution of metastatic occurrences. Paget postulates that tumor cells with metastatic potential, denoted as the “seed”, selectively interact with a receptive local environment, the “soil”. This interaction suggests the importance of a favorable microenvironment and positive compatibility between tumor cells and their surroundings [28]. Four decades later, James Ewing challenged the “seed and soil” model with the introduction of the “mechanical-circulatory” model and suggested that the appearance and spread of metastases are primarily influenced by mechanical forces and circulatory patterns. This viewpoint implies that metastases result from emboli of tumor cells, favorably entrapping in the initial organs connected via the circulatory network [42, 43].

Both Paget's and Ewing's theories support the concept of organ-specific metastases. Flow patterns can potentially elucidate why certain cancers tend to metastasize to particular organs while avoiding others [43]. For instance, prostate cancer frequently metastasizes to bones and mediastinal lymph nodes [44], suggesting that prostate cancer cells use both venous and lymphatic routes [45]. Similar patterns are observed in bone metastases from breast cancer [43]. Interestingly, about 40% of metastatic distribution can be attributed to blood circulation [46], with liver metastases notably influenced. Colorectal CTCs preferentially arrest in the liver due to the hepatic vascular architecture. Indeed, colorectal blood flow is drained by the hepatic portal system, whereas blood from the distal rectum goes directly to the lungs. This is why the lungs are a common site of liver cancer metastases [47]. However, this mechanism does not apply to highly vascular organs like muscles, spleen, or kidneys, which, despite their robust blood supply, remain infrequent sites of metastases. Conversely, organs such as bones and the brain are favored for metastases despite their lower blood flow rates [28].

According to the Paget's model [41], tumor cells might possess a strong affinity for certain remote secondary sites, offering a different explanation for organ-specific tendencies. For instance, primary mammary tumors predominantly metastasize to bones, lungs, liver, and the brain [48]. This might be influenced by the permissiveness of the tumor immune microenvironment for tumor growth and metastasis development. Myeloid cells, including macrophages and neutrophils, linked to tumor growth and progression [49, 50], are more abundant in lymphatic and bone metastatic lesions compared to primary breast lesions [51]. Hence, the native “soil” components play a role in metastatic organotropism, preparing the microenvironment and forming the premetastatic niche (PMN). Myeloid cells can be recruited to distant PMNs, enabling immune system suppression. Within this immunosuppressive context, studies revealed that normal stromal cells, mainly fibroblasts, can be reprogrammed within PMNs to facilitate metastasis. This reprogramming may be initiated by factors secreted by the primary tumor prior to the arrival of CTCs in the metastatic niche. These factors can be organ-specific, thereby influencing metastatic organotropism [28, 52].

In bone metastases, osteoblasts in the bone marrow can attract CTCs using chemoattractants like ligand stromal cell-derived factor-1 (SDF-1) or C-X-C motif chemokine 12 (CXCL12), which binds to C-X-C motif receptor 4 (CXCR4) receptors highly expressed in breast cancer cells. Matrix metalloproteinase 1 (MMP1) is also involved in this cellular extravasation and is part of the bone but also lung metastasis signature. Breast cancer can therefore metastasize to both bones and lungs [48, 53]. The diversity of breast cancer bone metastases, both osteolytic and osteoblastic, can be attributed to tumor-produced factors stimulating osteoclasts or osteoblasts, respectively. Among these factors, parathyroid hormone-related protein (PTHrP) and interleukin-11 (IL-11) play a pivotal role in osteoclastic bone resorption [54]. PTHrP-positive breast cancers exhibit a stronger tendency to metastasize to bones than PTHrP-negative ones [55].

Brain metastases require CTCs to cross the blood–brain barrier (BBB) first. Certain CTCs can permeate the BBB, entering the brain parenchyma [56]. Subsequent extravasation into the brain parenchyma is facilitated by mediators like cyclooxygenase 2 (COX2) and MMP2, also involved in lung metastasis formation [57]. In the brain, reactive or activated astrocytes contribute to cerebral homeostasis by releasing plasmin in response to CTCs, functioning as an antitumor response. CTCs counteract this by secreting plasminogen-activator inhibiting protein neuroserpin, promoting their survival [58]. Over time, the relationship between CTCs and the brain “soil” evolves, as activated astrocytes protect tumor cells from chemotherapeutic drugs, much like they do with neurons [59]. Additionally, metastatic cells can exploit astrocytes. For instance, IL-23 supports melanoma brain metastases’s progression and invasion, with melanoma brain metastatic cells upregulating MMP2-mediated IL-23 expression in astrocytes [60]. A subset of reactive astrocytes exhibits a signal transducer and activator of transcription 3 (STAT3) activity induced by tumor cells, which modulates immune responses, ultimately converting the naive “soil” into a tumor-promoting environment [61]. STAT3 also influences tumor migration, invasion, angiogenesis, and cell survival through elevated MMP-2, MMP-9, and EMT-related gene expressions, often activated by IL-11 [62].

The liver metastases in colorectal cancer are influenced by sustained STAT3 activation through sphingosine-1-phosphate receptor 1 (S1PR1) and IL-6, in conjunction with the recruitment of myeloid-derived suppressor cells (MDSCs) to the metastatic site [63]. Transmembrane emp24 trafficking protein 3 (TMED3) possibly mediates IL-11 secretion in hepatocellular carcinoma metastases [64], influenced mainly by hypoxia in various cancers [62].

Hypoxia characterizes the tumor microenvironment and is associated with modulating angiogenesis, vasculogenesis, and cancer progression by triggering factor production in stromal cells like VEGF or lysyl oxidase (LOX) [65]. VEGF receptor 1 (VEGFR1), the cognate receptor for VEGF, plays a critical role in PMN formation. VEGFR1 mediates the infiltration of bone marrow-derived cells (BMDCs) into the lung [66]. A similar mechanism applies to breast cancer lung metastasis colonization, where VEGFR1 influences metastasis-associated macrophages (MAMs) activation and the ensuing inflammatory response [67]. While this modulation supports the growth of metastatic nodules, it is not crucial for the de novo recruitment of BMDCs [66]. In terms of lung-PMN establishment, extracellular matrix (ECM) remodeling facilitates lung colonization by CTCs, often mediated by LOX. The involvement of LOX in PMN formation has been demonstrated, particularly after early-stage tumor resection, even before detectable metastases emerge [68].

**Fig. 1:**
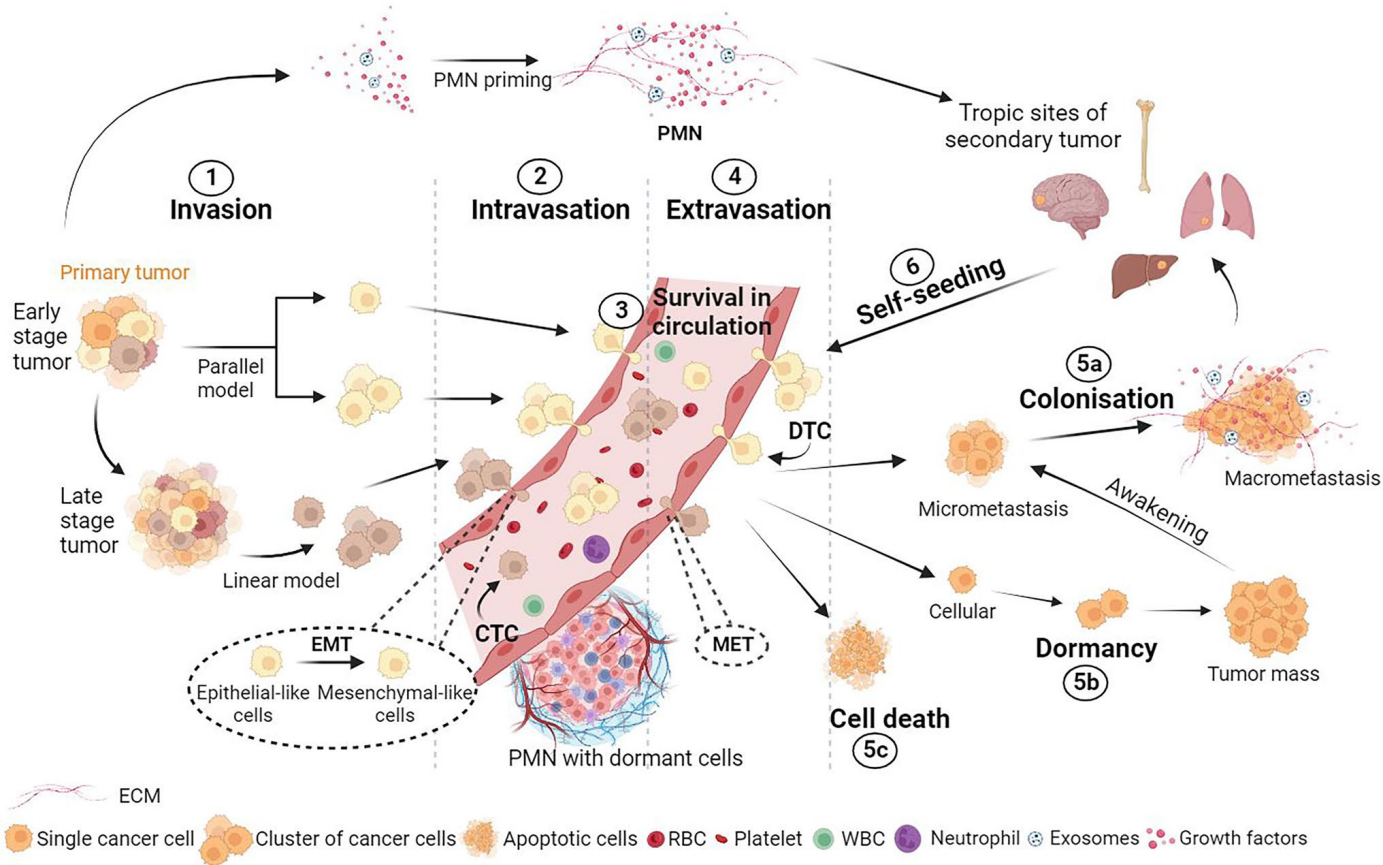
The metastatic cascade. (1) Metastasis is a multistep process, beginning with primary tumor growth and cellular transformation. Tumor cells can escape from the primary tumor at either early (parallel model) or late (linear model) stages of tumorigenesis. During the invasion step, tumor cells penetrate the surrounding host stroma. (2) Cells detach from the primary mass either as single cells or cluster of cells and subsequently acquire a dynamic phenotype through the EMT process, leading to their intravasation into the bloodstream. This step allows tumor cells to cross the endothelium and enter the circulation; these cells are called CTCs. (3) In the circulation, most of cancer cells die due to immune attack and physical damage caused by shear forces. Platelets in the bloodstream play a protective role by binding to CTCs, enabling them to survive. (4) Next, CTCs extravasate from the circulation to PMN preferentially, undergo MET, and are now called DTCs after this step. PMNs are formed prior to the arrival of DTCs by the primary tumor, which primes the host organ microenvironment by the means of exosomes, growth factors and ECM remodeling. Intrinsic and extrinsic factors of cells and the TME determine the fate of DTCs. Indeed, after extravasation, DTCs can grow into (5a) micrometastases and macrometastases, (5b) undergo dormancy and awaken after months or years of latency, or (5c) die. (6) Additionally, tumor cells have the capacity to re-enter circulation after extravasation at a distant site and return to seed the primary tumor. This is the concept of self-seeding. *CTC* circulating tumor cell, *DTC* disseminated tumor cell, *ECM* extracellular matrix, *EMT* epithelial to mesenchymal transition, *MET* mesenchymal to epithelial transition, *PMN* premetastatic niche, *RBC* red blood cell, *TME* tumor microenvironment, *WBC* white blood cell. Created with BioRender.com

Furthermore, tumors with specific genotypes tend to metastasize preferentially to define organs. This phenomenon establishes a link between tumor genotypes and metastatic organotropism, as seen in epidemiological studies. Different primary tumors with equivalent gene mutations often metastasize to the same secondary site [69], sharing common genetic alterations despite distinct primary origins (Fig. 1). The shared molecular features of these cells play a role in their adaptation to the same host microenvironment. However, the impact of a metastatic signature may vary depending on organotropism [70]. The ability to predict primary tumor organotropism would significantly benefit patients, enabling more targeted screening, timely follow-up, and potentially proactive treatment. Gerratana et al. proposed an approach utilizing machine learning algorithms and liquid biopsies to obtain insights into potential metastasis sites. Central to this approach is the enumeration of CTCs and the analysis of cell-free DNA (cfDNA), which are nucleic acids detectable in fluids and provides information on both primary and secondary tumors [71].

## Cell-free DNA (cfDNA)

### Description of cfDNA

The concept of cfDNA was initially introduced by Mandel and Metais in 1948 [72]. It constitutes a portion of circulating nucleic acids (CNA) in the forms of single-stranded DNA (ssDNA) and double-stranded DNA (dsDNA). Found in various bodily fluids, cfDNA circulates within vesicles like exosomes, microvesicles, or apoptotic bodies, as well as in complex molecular structures, such as nucleosomes, virtosomes, NETs (neutrophil extracellular traps), and eosinophil extracellular DNA traps (EETs). Additionally, it can also bind to serum proteins or be situated outside cell membranes. Importantly, cfDNA is protected from degradation by nucleases and escapes immune system detection. Current understanding acknowledges multiple sources of cfDNA, including processes like oncosis, necrosis, apoptosis, phagocytosis, and active secretion [73]. Notably, even healthy non-tumoral cells such as stromal cells, endothelial cells, lymphocytes, and other immune cells can release cfDNA. In cancer patients, cfDNA originates from three cellular sources: normal non-tumor cells, malignant tumor cells, and cells within the tumor microenvironment [73, 74]. Additionally, viral nucleic acids can also be identified in the bloodstream [75]. A subset of cfDNA known as ctDNA emerges from tumor cells [76] and comprises cancer-specific somatic mutations, epigenetic alterations, and chromosomal aberrations [77].

Apoptosis is widely recognized as the primary source of cfDNA [76], with this process essential for cellular balance and turnover. It is controlled by pro-apoptotic and pro-survival B-cell lymphoma 2 (BCL-2) family proteins and triggers a caspase-dependent proteolytic cascade that leads to DNA fragmentation, producing fragments approximately 180 bp long [76, 78], and 145 bp for ctDNA [79]. These apoptotic cell-released cfDNA fragments are enclosed in nucleosomes [78, 80] that provide protection and stability [11]. The presence of nucleosomes correlates significantly with blood DNA concentration [81]. These nucleosomes can be packaged into apoptotic bodies when membrane blebbing occurs and subsequently phagocytosed by macrophages or dendritic cells [11]. However, apoptotic bodies are not the primary vehicles of cfDNA, as demonstrated in a rat cancer model where cfDNA detection at early cancer stages did not involve apoptotic bodies [82]. Necrosis represents a passive mode of cfDNA release, producing larger DNA fragments (over 1000 bp) [78]. Active secretion is another potential cfDNA release mechanism [83], exemplified by virtosomes-nucleic acid-lipoprotein complexes released by living cells, which can be taken up by other cells [78]. Living cells also secrete EVs like exosomes through the exocytosis of multivesicular bodies (MVBs) [84]. While the role of exosome-conveyed cfDNA in blood is not fully understood [78], it is recognized that exosomes participate in cell signaling and intercellular molecular communication upon internalization by other cells [85]. Furthermore, the concentration of exosomes is notably higher in the blood of cancer patients compared to healthy individuals [86]. Some investigations have suggested CTCs as potential sources of cfDNA, but their abundance does not correlate with cfDNA concentration in blood [73, 80]. Another mechanism for cfDNA release is NETosis, an independent process involving active neutrophils releasing NETs [87]. Conditions like sterile inflammation, infection, or hypoxia can trigger NETosis [87, 88]. Predominantly composed of cfDNA, the degradation of NETs contributes to elevated cfDNA levels in various diseases [78].

### Concentration of cfDNA and tumor progression

Numerous studies have highlighted elevated levels of cfDNA in cancer patients as compared to healthy individuals. Notably, the concentration of germline cfDNA originating from healthy cells remains stable, in contrast to ctDNA levels linked to tumor cells [73]. Moreover, the activity of DNase is reduced in the plasma of cancer patients in comparison to healthy subjects [89, 90]. Furthermore, cfDNA concentrations are increased in patients with advanced or metastatic cancer compared to those with early-stage or non-metastatic cancer, respectively. Consequently, as cancer progresses and metastases become evident, cfDNA levels tend to rise [73]. According to the study of Lin et al., cfDNA concentration in the blood displays no correlation with age, gender or cell proliferation, but is significantly associated with the tumor size and the tumor, node, metastasis (TNM) staging [91].

Following surgical interventions and/or chemotherapy, there is a decrease in cfDNA levels, potentially reverting to pre-treatment levels. The initiation of chemotherapy can lead to heightened levels of nucleosomes and cfDNA due to treatment-induced apoptosis [92]. However, elevated cfDNA levels post-treatment are linked to poor prognoses and might signify an inadequate response to treatment, particularly in individuals with metastases [93]. Eastley et al. found cfDNA in patients with soft tissue sarcoma (STS) without metastases at diagnosis and who were radiologically considered “disease-free” This occurrence could be attributed to the existence of micrometastases that elude radiological or clinical detection, subsequently releasing cfDNA [94]. It is widely admitted that cfDNA has the potential to serve as a biomarker for MRD detection in solid tumors [95]. For instance, in patients with medulloblastoma, Escudero et al. detected cfDNA in individuals who exhibited a complete response three months prior to radiological relapse, laying emphasis on the potential of cfDNA for MRD detection [96].

### cfDNA and tumor genomic landscape

cfDNA originating from cells undergoing apoptosis offers insights into therapy-responsive cells, while cfDNA from viable cells reveals information about therapy-resistant cells [97]. Notably, specific mutations linked to chemotherapy resistance and tumor development can be discerned in cfDNA, often months before clinical or imaging-based progression becomes evident. This advantage over traditional tissue biopsy arises from the ability to perform the liquid biopsy without imaging [80, 97]. Analysis of cfDNA provides a comprehensive perspective on genetic alterations found in both primary tumors and metastatic sites, potentially reflecting the evolutionary course of the tumor [80, 92]. Numerous studies have demonstrated the congruence between genomic changes detected in tumor tissues and cfDNA. Adalsteinsson et al. highlighted a robust alignment between mutational signatures and neoantigens within cfDNA and those found in corresponding solid biopsies [98]. Furthermore, it has been verified that cfDNA captures the clonal diversity present in primary tumors and metastases, utilizing both tumor sampling and RepSeq approaches [99, 100]. In certain instances, genomic profiling of cfDNA uncovers mutations absent in tumor tissue analyses, and vice versa [100]. Discrepancies between cfDNA and older primary tissue biopsies could arise due to somatic evolution and clonal shifts during tumor progression or post-chemotherapy [101, 102]. Additionally, the presence of somatic clones detected in cfDNA might not originate from the tumor but from clonal hematopoiesis (CH) [103]. Colorectal cancers, for instance, exhibit intratumor heterogeneity from the early stages, often not bound by clonal selection. Sottoriva et al. propose that the malignancy potential of certain tumors emerges quite early, particularly those with variegated alterations [104]. Notably, in cases where primary colorectal tumors exhibit significant heterogeneity, the likelihood of developing liver metastases increases [105]. DNA alterations driving oncogenesis identified in cfDNA correlate with those seen in corresponding metastatic tissues [106], with cfDNA surpassing a single tumor biopsy in detecting heterogeneous driver alterations [107]. The sequence of genetic alterations and the presence of oncogenic drivers significantly influence tumor progression [108, 109]. As a result, genetic heterogeneity complicates the precise identification of somatic mutations within cfDNA. However, it offers a reliable portrayal of the tumor genomic landscape and its real-time clonal evolution.

### Functions of cfDNA and “genometastasis”

In normal cells, the release of cfDNA serves the purpose of discarding damaged nucleic acids, primarily accomplished through exosomes. This process is pivotal in upholding cellular equilibrium and genomic integrity [110, 111]. Exosomes, in addition, hold significance in intercellular communication and the maintenance of cellular homeostasis [111, 112]. Tumor-derived EVs, particularly exosomes, can play a role in ECM remodeling [113] and in PMN remodeling, inducing macrophage polarization in M2 state, an immunosuppressive microenvironment and stimulating angiogenesis [52]. These EVs can be used to predict organotropism, as specific organ metastases are associated with exosomal integrins such as α6β4 and α6β1 for lung metastases, and αvβ5 for liver metastases [114]. Tumor-derived exosomes further promote organ-specific metastatic colonization, particularly adipocyte-derived exosomes activating signaling pathways that enhance the metastatic capacity of tumor cells. For instance, adipocyte-derived exosomes originating from breast cancers drive lung metastases [115], while those from epithelial ovarian cancers facilitate peritoneal metastases. These exosomes are released by mature adipocytes harboring mesenchymal stem cell (MSC)-like characteristics [116]. It is now accepted that MSC-derived exosomes can switch the phenotype of stromal or normal cells to a malignant one by delivering their cargo (nucleic acids and proteins) into recipient cells (including fibroblasts, endothelial cells, epithelial cells and infiltrating leukocytes) [117]. In fact, MCSs can differentiate into CAFs via the TGF-β/Smad pathway activated by tumor-derived exosomes. By influencing the surrounding ECM that promote metastasis spread, the role of CAFs in PMN formation is significant [118]. Additionally, CAFs can transform epithelial cells into a pre-malignant phenotype in the presence of tumor-derived exosomes [119]. The interaction between exosomes and recipient cells is mediated through cfDNA within exosomes [78], which acts as an intercellular messenger [73].

**Fig. 2:**
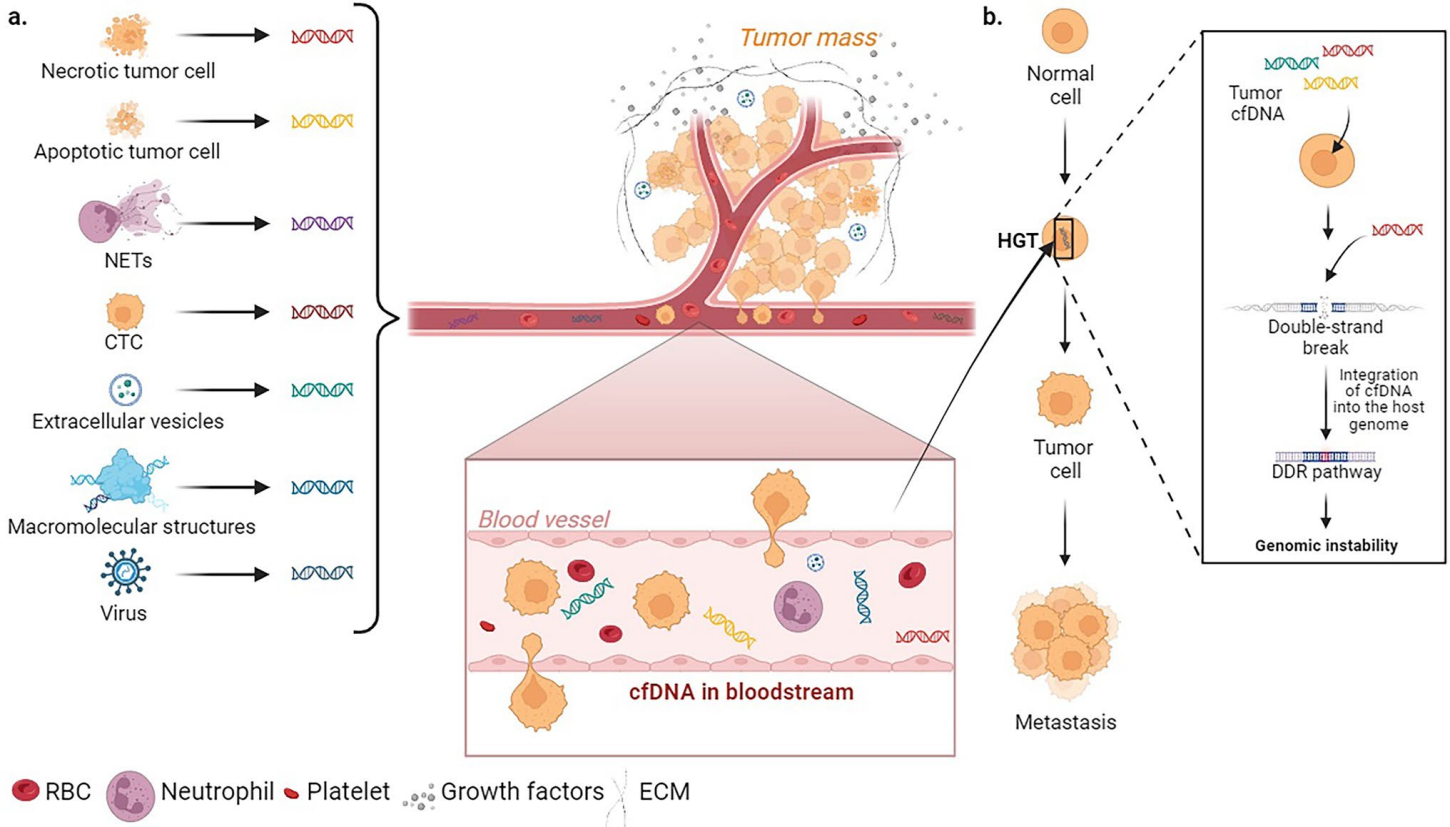
The genometastasis theory. **a** Sources of cfDNA. The population of cfDNA in bloodstream is heterogeneous due to various mechanisms by which cfDNA is released. Different cellular sources contribute to this heterogeneity: necrotic and apoptotic tumor cells, NETs, CTCs, exosomes and other EVs, macromolecular structures (virtosomes), and viruses. **b** Metastasis formation. Among biological functions of cfDNA, the oncogenic transformation of susceptible cells by horizontal transfer provides another explanation of metastasis formation besides the traditional metastatic cascade. This is the putative “genometastasis” theory, according to which nucleic acids released into the circulation are able of transfecting susceptible cells via the horizontal gene transfer (HGT). After entering the nuclei of healthy cells, the cfDNA from tumor cells integrate into their genome following activation of a cellular DDR, resulting in the malignancy of these transformed cells due to the genomic instability generated. *cfDNA* cell-free DNA, *CTC* circulating tumor cell, *DDR* DNA damage response, *ECM* extracellular matrix, *EVs* extracellular vesicles, *HGT* horizontal gene transfer, *NETs* neutrophil extracellular DNA traps, *RBC* red blood cell. Created with BioRender.com

Once entering into normal cells, cfDNA integrates host cells genome, provoking biological responses such as DNA damage, mutagenesis, or even apoptosis in the recipient cells [120]. This phenomenon is known as horizontal gene transfer (HGT), where fragments of DNA can be transferred horizontally or even vertically between cells [121]. Hence, cfDNA can also be considered such as a mobile genetic element [120]. Studies suggest that cfDNA shed by tumors into the bloodstream contributes to the malignant transformation of non-tumor cells, along with tumorigenesis and metastasis development [122]. This postulate constitutes the basis of the “genometastasis” concept introduced by García-Olmo et al*.* in 2000s, which describes the ability of ctDNA to transfect distant cells and form metastases (Fig. 2) [8, 10]. The integration of ctDNA into neighboring normal cells via the DNA-damage-repair (DDR) pathway remains an unclear process [123]. However, there is evidence supporting this concept, including the oncogenic transformation of cultured cells by cell-free plasma from cancer patients, particularly particles smaller than 0.4 µm in diameter, potentially exosomes or apoptotic bodies containing nucleic acids [124].

These tumor-derived exosomes carrying oncogenic drivers can transfer malignant traits to recipient cells, even reprogramming normal fibroblasts into different types of cancer cells [125]. Apoptotic bodies, also part of the process, can be phagocytosed by recipient cells leading to their oncogenic transformation [126]. Ehnfors et al*.* have demonstrated the phagocytosis of apoptotic bodies containing tumor DNA by endothelial cells and fibroblasts, endorsing changes that allow them to acquire tumor features [127]. The virtosome, another carrier of cfDNA, is capable of transfecting cells. After penetrating neighboring cells, virtosomes modify the cells genetic program, promoting tumor development [83]. In fact, this virtosome-mediated transfection is the foundation of the concept of transcession that Anker *et* Stroun developed before the discovery of virtosomes [128, 129]. It constituted the first proof of HGT [73]. Overall, research about genometastasis focus on DNA transfer between two distinct entities. However, in the case of metastases, the transfer occurs within the same organism. Using the term “HGT” appears unsuitable for DNA transfer between cells within the same entity, as it refers to DNA fragments rather than whole genes. Thus, Thierry et al. coined the term intra-organism genetic transcession (IGT) to describe this process [73].

All of vesicular structures such as apoptotic bodies or exosomes provide stability to oncogenic fragments. In this context, Antonyak et al. suggest that these EVs contribute to transforming stromal cells, fibroblasts, and epithelial cells within PMNs into a tumor phenotype [130], especially when the ctDNA they carry includes oncogenic drivers as H-*ras* which stimulates cell proliferation [131]. Nonetheless, this theory does have its limitations. Efficient secretion of tumoral EVs necessitates continuous release and acidic, hypoxic conditions [52]. Additionally, the effectiveness of HGT depends on the uptake capacity of recipient cells [132], which may render certain cells refractory to transformation [133]. Although this concept has been clearly demonstrated in vitro and is interesting from a biological standpoint, there is currently no clinical evidence to substantiate the genometastasis theory in in vivo settings or in pre-clinical models.

Furthermore, cfDNA plays a role in immune responses and blood coagulation through a process called NETosis [134]. NETs, implicated in both innate immunity [135] and the inflammatory state of cancer, contribute to thrombosis, tumor cell proliferation, and metastases [136]. NETs engage in cross-talk with platelets and CTCs, impacting thrombosis, inflammation [137], cancer progression, and metastasis development [138, 139]. The principal component of NETs, cfDNA, exhibits procoagulant [87] and proinflammatory properties [110]. NETosis contributes to creating a favorable microenvironment for tumor and metastasis growth, particularly at sites with accumulated neutrophils [140]. Tumor-derived exosomes also influence this process by stimulating NETosis [136]. Different components of NETs, especially DNA, play a role in metastasis formation. DNA binding to the transmembrane protein CCDC25 enhances the attraction of CTCs to distant sites, facilitating metastatic spread [141]. In addition, like exosomes in ovarian cancer, NETs facilitate metastatic formation to the omentum [142].

## Discussion

Over the past century, the works of Ewing [3], Fidler [12], Paget [41], Weiss [42] and others regarding metastasis formation have been extensively studied and validated, gaining in widespread acceptance. In essence, the development of secondary tumors hinges on the success of each step of the metastatic cascade. This process is influenced by intrinsic features of tumor cells and the host microenvironment, which can be summarized as the three S’s: selective, sequential, and stochastic [143]. Nevertheless, this metastatic cascade is overall an inefficient stepwise process, with survival of CTCs within the bloodstream constituting the most critical step for successful metastatic spread [144]. Furthermore, the suitability of the host microenvironment for metastatic growth constitutes an additional hurdle in the metastatic cascade. Indeed, the creation of a “metastatic niche” prior to the arrival of CTCs seems to be a key step in the metastatic cascade. This concept, known as PMN, is initiated by the primary tumors, which can secreted factors that can be organ-specific, thereby influencing metastatic organotropism [52]. Among these factors, exosomes and NETs play essential roles in PMN remodeling and both are associated with cfDNA in bodily fluids, although cfDNA can also exist independently [110]. Interestingly, some studies have shown that this cfDNA, especially oncogene fragments released by tumor masses, could play a transformative role in metastases development, serving as a testament to the “genometastasis” theory [10]. This concept posits that cells already present in the host organ undergo oncogenic transformation via cfDNA secretion from the primary tumor, but it does have its limitations and lacks in vivo evidence. As a result, this theory completely contradicts the decades-old assertion that metastases are the consequence of the spread of CTCs from the primary tumor. However, the “genometastasis” theory and the metastatic cascade statement are not mutually exclusive. Since the metastatic cascade is inefficient, the proliferation of DTCs cannot exclusively account for metastasis development. CTCs, though not the sole cause of metastasis, are indicative of a poor prognosis, particularly in early cancer stages [145]. The notion that metastasis may arise from the abnormal expansion of normal cells transformed by cfDNA into a metastatic niche is quite appealing. In vitro studies have demonstrated the malignant transformation of non-tumoral cell lines using sera from cancer patients [133]. Nonetheless, direct in vivo evidence regarding the metastatic potential of cfDNA is scarce but several studies proved that tumor-derived exosomes can prepare the PMN by reprogramming resident cells [119]. Furthermore, cfDNA and CTCs may collaborate in metastasis formation. Indeed, Trejo-Becerril et al. hypothesized that ctDNA can induce the outgrowth of micrometastases via HGT of micrometastatic cells, resulting in the formation of macrometastases. This highlights the potential contribution of ctDNA to tumor progression in vivo [146] and the potential collaboration between cfDNA and CTCs in the metastasis formation. Notably, cfDNA is not an inert element and can act as a mobile genetic element [147] among other extensively demonstrated functions [148].

**Fig. 3:**
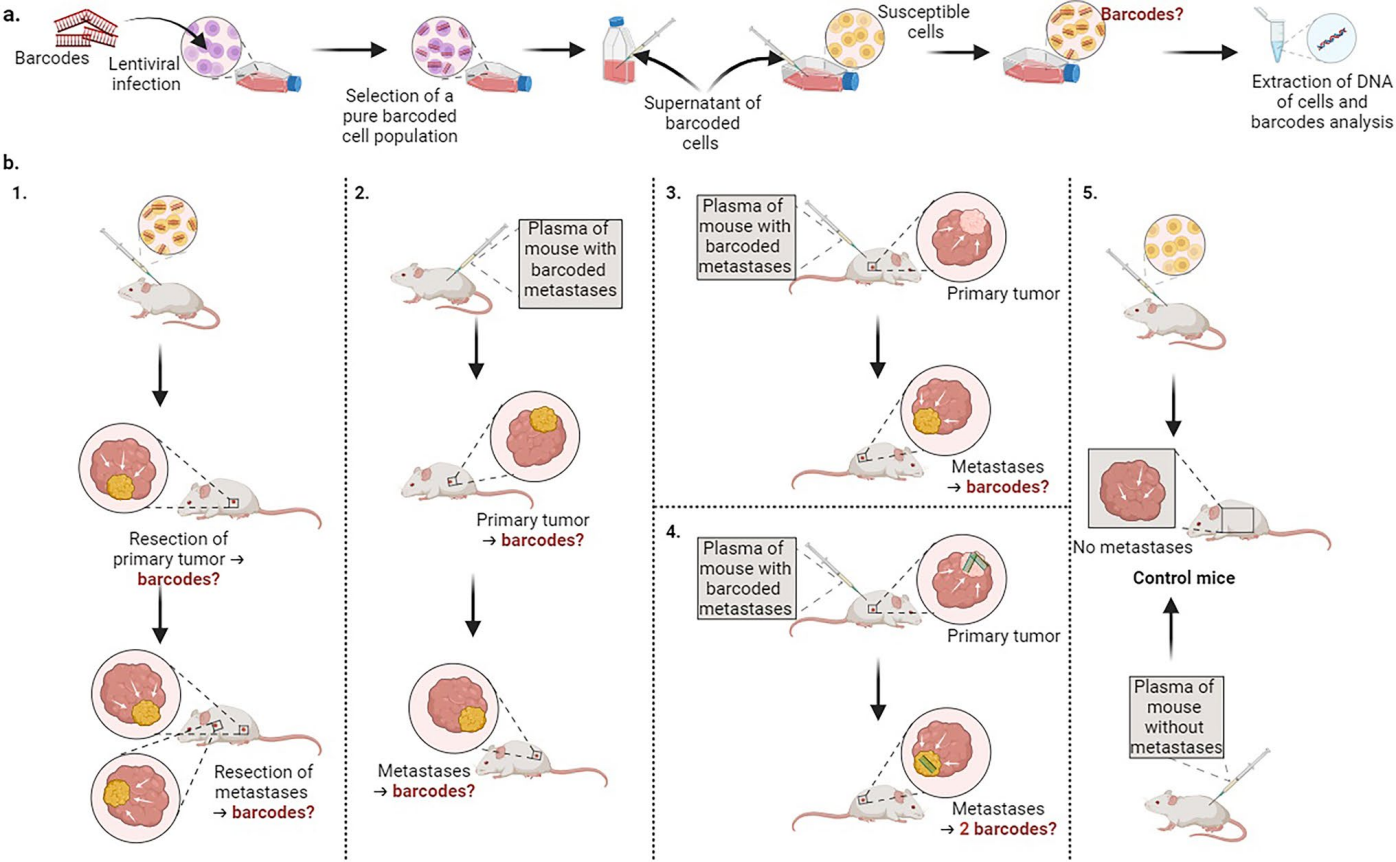
Schematic proposition of an experimental workflow to study the different theories of metastasis formation. **a**. In vitro process. Barcodes are transfected into a cell line with high metastatic potential (purple cells) by lentiviral transfection. After a few days of growth, cells with barcodes are selected to obtain a pure barcoded cell population. Next, the supernatant of these barcoded cells is harvested and a new cell line without metastatic properties (yellow cells) is cultured with the supernatant of these barcoded cells. Then, cells are sorted and analyzed. The barcodes should be integrated into the yellow cells. **b**. In vivo process. (1) Barcoded yellow cells are injected in a tumor-free mouse. Following resection of primary tumor and metastases, cells are analyzed and we predict that tumors contain barcodes, thus demonstrating that the metastatic behavior of purple cells has been transmitted to yellow cells by horizontal transfer via the supernatant. (2) Plasma from the mouse with metastases containing yellow barcoded cells is injected into a tumor-free mouse. Cells from primary tumor and metastases are analyzed and we predict that both tumor types contain the barcodes of yellow cells, which confirm the T2 theory. (3) The experiment in (2) is replicated in a mouse with a pre-existing primary tumor and we expect the metastases to contain the plasma barcodes or not. (4) The experiment in (2) is replicated in a mouse with a pre-existing primary barcoded tumor and we expect the metastases to contain barcodes from both the primary tumor and those found in plasma, proving the T3 theory. (5) Control mice receive injections of unmodified yellow cells and plasma from a tumor-free mouse. No metastases will appear in this model. Created with BioRender.com

Taking into account all relevant facts, we present three proposed mechanisms for the development of metastasis. The first theory (T1), which has extensively been studied, states that metastases originate from CTCs and requires no further substantiation; instead, clarification is needed on which clones specifically result in metastasis. The second theory (T2), corresponding to the “genometastasis” theory, suggests that metastases are the result of cells transformed by ctDNA or exosomes. Lastly, the third (T3) proposes a synergistic collaboration between CTCs and ctDNA, both originating from the primary tumor, as the cause of metastases (Fig. 3). To determine the origin of metastases, several protocols can be proposed. The first approach is to track the division and dissemination of cells from the founding clone, the most recent common ancestor, and to investigate in what extent metastases are the result of cell dissemination from the primary tumor, in line with the metastatic cascade statement. This is also called the tumor lineage tracing. To this end, the use of molecular barcoding is intended. Barcodes consist of short randomized sequences of DNA and are integrated into the genome. Barcoding of cells has already been developed to assess the clonal evolution of human cells in murine xenograft models [149], but also to evaluate clonal responses to anti-cancer drugs and to monitor treatment effects [150]. Hesin et al*.* validated the double barcoding of a subpopulation of clones to explore the formation of primary tumors and metastases in animals, as well as the effect of anti-cancer drugs on the number of clones. In their study, the two cell lines have the same capacity of metastasize to the lung and liver [151]. Echeverria et al. obtained similar results, founding the same dominant barcodes in brain, liver and lung metastases, in accordance with the metastasis formation from same subclones of the primary tumor. Indeed, less than 5% of primary tumor barcodes are detected in all metastases. However, dominant clones are present in smaller quantities than seeding clones. A large proportion of seeding clones is shared with other metastases but not with primary tumor [152]. This phenomenon may be explained by the possibility that a metastasis can metastasize and consequently seed another PMN, similar to the self-seeding capabilities of metastases. Taken together, these results support the theory of CTCs being the origin of metastasis (T1), but only specific clones of the primary tumor may successfully seed secondary organs.

Efforts to confirm the three proposed theories could involve using molecular barcodes coupled with fluorescent proteins. This entails infecting high-metastatic potential cell lines (*e.g.,* the KM12 cell line) with lentiviral particles containing barcodes, followed by exposing susceptible cells to conditioned medium from barcoded cells. Cells can be expected to be modified by nucleic acids present in the supernatant, as they integrate the barcodes, and acquire the behavior of barcoded cells. This in vitro transformation of cell lines with supernatant of tumor cells has already been demonstrated, such as the *KRAS*^*G12D*^ oncogene, which can be transferred by HGT of cfDNA from colorectal tumor cells into liver cells, mimicking the phenomenon of colorectal metastasis formation [153]. The use of barcodes would provide novelty and evidence that cells are transformed by a component present in the conditioned medium secreted by tumor cells, in line with T2 theory. Moreover, the injection of barcoded cell lines in a xenograft mouse model followed by monitoring of metastasis progression has already been performed [154]. Based on these results, we propose an experimental model in which cultured cells previously conditioned with supernatant of barcoded tumor cells would be transplanted into mice. This model will prove that the ability of cells to form tumor can be transmitted by HGT via the supernatant, in addition to the preparation of PMNs by exosomes, which are present in the supernatant of cultured cells in vitro and in plasma in vivo [155]. Control mice will be transplanted with the same normal cell line without modification. We anticipate that mice transplanted with modified cells will develop metastases, whereas those transplanted with unmodified cells will not. In addition, injection of plasma from mouse with barcoded metastases into a tumor-free mouse can also be considered. We propose using a second mouse with a primary tumor without metastases, and injecting this plasma into the second mouse as well. We expect the barcodes to be identifiable in tumors of the first group of mice, and the emergence of barcoded metastases at a later stage. In the second group of mice, it is believed that metastases contain barcodes. If the same experiment is performed in a mouse with a primary tumor generated with barcoded cell line, we can also assume that both types of barcodes will be found in the metastases, according to the T3 theory.

One feature limiting the confirmation of the T2 theory with this experimental model is the choice of organs from which the cell lines used in experiment are derived. In fact, some studies on HGT failed to demonstrate malignant transformation of normal cells, whereas in others cells this has been an evidence, even within the same experiment [153]. Indeed, the cells must be receptive to the transforming agent otherwise no modification seems possible. Arena et al*.* suggest that oncogenic transformation occurs in organs from the same embryological layer, providing an explanation for the metastases organotropism. Furthermore, cells may harbor a mutation in an onco-suppressor gene or an oncogene to facilitate the integration of the transforming agent [156]. In essence, the intricate nature of metastasis development involves factors such as organ origin and the susceptibility of recipient cell. While the idea of metastasis originating from CTCs or the transformation of normal cells via nucleic acids presents two opposing theories, they are not mutually exclusive. The presence of CTCs and the transformation of normal cells both contribute to metastasis, with the exact balance and interplay between these processes warranting further exploration.

## Conclusion

The established theory of metastasis formation revolves around the concept of the metastatic cascade. While the intriguing “genometastasis” theory offers potential explanations for phenomena unaccounted for by the current model, the lack of in vivo evidence necessitates numerous experiments to elucidate its validity. Nonetheless, an alternative hypothesis suggests a collaborative role of CTCs and cfDNA in metastasis formation, with each potentially raising the metastatic potential of the other. Exploring the factors contributing to metastatic genesis and understanding the mechanisms underlying metastatic organotropism holds promise for curbing the progression of metastatic disease and improving its prevention.
